# Detection of FeChPV in a cat shelter outbreak of upper respiratory tract disease in China

**DOI:** 10.3389/fmicb.2022.1064747

**Published:** 2022-12-08

**Authors:** Xiangqi Hao, Yanchao Li, Bo Chen, Hanhong Wang, Xinrui Wang, Xiangyu Xiao, Pei Zhou, Shoujun Li

**Affiliations:** ^1^Guangdong Provincial Key Laboratory of Prevention and Control for Severe Clinical Animal Diseases, College of Veterinary Medicine, South China Agricultural University, Guangzhou, China; ^2^Guangdong Provincial Pet Engineering Technology Research Center, College of Veterinary Medicine, South China Agricultural University, Guangzhou, China

**Keywords:** feline chaphamaparvovirus, outbreak, cat shelter, upper respiratory tract disease, viral encephalitis

## Abstract

Feline parvovirus often causes a fatal infectious disease and has a serious impact on domestic cats and wild felines. Feline chaphamaparvovirus (FeChPV) is a novel type of feline parvovirus that has been successively identified in Canada, Italy, and Turkey. The prevalence and pathogenicity of FeChPV in other regions is still unknown. In this study, we recorded the detection of FeChPV in a cat shelter in China. A high prevalence (81.08%, 30/37) of FeChPV was detected in cats with symptoms of upper respiratory tract disease (URTD) in this cat shelter. Multiple pathogen testing indicated high coinfection rates of 80% (24/30) with other common viruses in FeChPV-positive cats. Analyses of the necropsy and histopathological findings revealed severe lymphadenitis, encephalitis, and viral DNA in several tissues (including brain) of the deceased cat. Finally, we obtained nearly full-length genomes of four strains with 98.4%~98.6% homology with previously reported genomes. Notably, VP1 proteins showed seven unique amino acid mutations, while NS1 proteins carried eight mutations. In the evolutionary tree based on VP1 and NS1, the sequences clustered in a large branch with Italian and Canadian FeChPV strains. Given the possible association of FeChPV with URTD, further studies are necessary to evaluate the pathogenicity and epidemiological characteristics of this novel feline pathogen.

## Introduction

Members of the *Parvoviridae* family are small, nonenveloped viruses with a linear single-stranded DNA genome of 4–6 kb (Cotmore et al., 2019). An increasing number of novel parvoviruses have been identified, and thus far, the *Parvoviridae* family comprises three subfamilies: *Parvovirinae*, *Hamaparvovirinae*, and *Densovirinae* (Penzes et al., 2020; Abayli and Can-Sahna, 2022). *Hamaparvovirinae* has a broad host range, including vertebrates and invertebrates (Liu et al., 2022). Chaphamaparvovirus (ChPV), a genus in *Hamaparvovirinae*, has been reported to have a large host range, including mammals, such as dogs, cats, black bears, rats, mice, sheep, and pigs (Yang et al., 2016; Alex et al., 2020; Ge et al., 2020; Wang et al., 2020; Di Profio et al., 2022); birds, such as barn owls, peafowl, paradise tanagers, red-crowned cranes, parrots, ducks, and chickens (Lima et al., 2019; Wang et al., 2019; Liu X. et al., 2020; Vibin et al., 2020; Hargitai et al., 2021; Sarker, 2021); and even fish, such as tilapia (Du et al., 2019).

In 2019, feline Chaphamaparvovirus (FeChPV) was first identified in fecal samples from cats in an animal shelter system in Canada and was thought to be associated with an outbreak of diarrhea and vomiting in cats (Li et al., 2020). From 2019 to 2020, FeChPV was identified in Italian cats with upper respiratory disease and diarrhea. The detection rate of FeChPV was high (36.8%, 14/38) in clinical cases. Its positivity rate was higher than that of feline panleukopenia virus (FPV; 23.7%, 9/38), feline coronavirus (5.3%, 2/38), feline kobuvirus (5.3%, 2/38), and noroviruses (5.3%, 2/38). FeChPV was the most common enteric viruse and was thought to be associated with acute gastrointestinal diseases. However, there was no significant association between FeChPV and URTD, and the detection rate in respiratory samples from cats was low (3.3%–4.3%; Di Profio et al., 2022). Additionally, the virus was detected in the feces of healthy cats in Turkey in 2022 (Abayli and Can-Sahna, 2022). To date, the pathogenic role of FeChPV and the prevalence of the virus in other wild carnivores are still uncertain. ChPV was originally identified from oropharyngeal swab samples of fruit bats (*Eidolon helvum*) from Ghana (Africa) by metagenomic studies (Baker et al., 2013). Subsequently, similar viruses were also found in dogs and cats (Di Profio et al., 2022). Furthermore, it is noteworthy that in a previous study from China, a ChPV (named cachavirus-cat 1 and -cat 2) was found in the feces of diarrheic cats. The virus is closely related to the genetic signature of canine chaphamaparvovirus (CaChPV) with affinities up to 91.9%–97.0%. However, the homology of cachavirus-cat1 and -cat2 with FeChPV in cats is only approximately 73.2%–74.8% (Ji et al., 2020). ChPV of both canine and feline origin are currently classified as Carnivore chaphamaparvovirus 1 (CaChPV-1; Penzes et al., 2020; Di Profio et al., 2022). The above studies suggest that CaChPV-1 may have genotypic diversity and host diversity.

The genome of FeChPV is approximately 4,225 bp in length and includes three open reading frames, nonstructural protein 1 (NS1, 1977 bp), virion protein 1 (VP1, 1,527 bp), and nuclear phosphoprotein (NP, 561 bp). Phylogenetic analysis indicated the closest affinity between FeChPV and CaChPV (Li et al., 2020).

In this study, we described the first detection of FeChPV in a cat shelter in Guangzhou, China, at the end of July 2022. Viruses were detected in eye secretions and nasal swabs of cats with upper respiratory tract disease (URTD). Viral DNA was detected in multiple tissues; the diseased cats may die from viral infection, and several tissues were collected for pathologic analysis. In addition, nearly full-length genomes of four strains were sequenced, and data on the genetic characteristics of these viruses were analyzed. Further studies are needed to characterize the occurrence and distribution of FeChPV worldwide, with the aim of determining whether there is a risk of global transmission.

## Materials and methods

### Sampling

We recorded an outbreak of a suspected influenza-like outbreak in a cat shelter containing 37 cats in Guangzhou, Guangdong Province, on 29 July 2022. The background information (including gender and breed) of the cats is listed in Supplementary Table S1. At first, only a few cats showed obvious clinical signs, such as fever, cough, purulent nasal discharge, and purulent eye discharge, but no cats died. The sick cats were treated with antibiotics and interferon-ω, but the outbreak was not mitigated, and the disease quickly spread among other cats in the shelter. Finally, ocular and nasal secretions from all cats were collected and preserved in prechilled DMEM. Samples were rapidly transported to the laboratory for the detection of common feline viruses.

As the disease progressed, some cats showed symptoms such as loss of appetite, depression and head tilt. Overall, a total of five cats in the shelter died. One dead cat (#7, only FeChPV detected) was necropsied, and tissue samples were collected for pathological analysis.

### Nucleic acid extraction and real-time PCR

Ocular and nasal swabs from the cats were immersed in DMEM, vortexed and then centrifuged (12,000 rpm) at 4°C. Subsequently, 200 μl of supernatant was used for DNA/RNA extraction using the DNA/RNA Extraction Kit (Magen, Guangzhou, China). Then, according to previous studies (Hao et al., 2021), the RNA was reverse transcribed into cDNA. FeChPV was first detected (Di Profio et al., 2022). Then, screening for common respiratory pathogens, including influenza A virus (IAV), feline calicivirus (FCV), feline herpesvirus-1 (FHV-1), FPV, *Mycoplasma felis* (*M. felis*) and *Chlamydia felis* (*C. felis*), with reference to previous methods (Pei et al., 2020), was also performed. The primers for the detection of the common respiratory pathogens are listed in Supplementary Table S2.

To isolate viral DNA from tissue samples, each tissue sample (1 g; including heart, liver, spleen, lung, kidney, brain, cervical lymph node, nasal turbinate, trachea, stomach, duodenum, jejunum, ileum, cecum, and colon tissues) was first homogenized in phosphate-buffered saline (1 ml) using a homogenizer. Referring to previous studies (Liu et al., 2022), the virus DNA loads of FeChaPV were determined using the SYBR Green I method (AbColonal, China) on an LC480 instrument (Roche). The primers were synthesized by Sangon Biotech (Shanghai, China) and are shown in Table 1.

**Table 1:** List of primers and ISH probes used for FeChPV.

Oligonucleotide	Sequence (5′–3′)	Sense	References	Use
FeChPV_F	GGTGCGACGACGGAAGATAT	+	Li et al. (2020)	Diagnostic PCR
FeChPV_R	CAACACCACCATCTCCTGCT	−	Li et al. (2020)	Diagnostic PCR
FeChPV_qF	GCGTATACCGTATGGGGTCA	+	Liu et al. (2022)	qPCR
FeChPV_qR	AGTCCCTGGGAATCTCCATC	−	Liu et al. (2022)	qPCR
FeChPV_NS1F	ATGGAACGTAGCAGACGTGCT	+	This study	Genome sequencing
FeChPV_NS1R	TCAGCCATATTGTTTCTGCAAATAAGACA	−	This study	Genome sequencing
FeChPV_VP1F	ATGTTCCTACTAAAACTGATTGGTTG	+	This study	Genome sequencing
FeChPV_VP1R	TTATTTGTCATCTTCTTCGATCATTTC	−	This study	Genome sequencing
FeChPV_probe 1	GCTCTCGCTGCGGTTTCTGTAAGT	−	This study	ISH
FeChPV_probe 2	TTCGCTGTCATCAATAGTCTCGCA	−	This study	ISH
FeChPV_probe 3	CGCACCCGCTAATCTGTCTACTTC	−	This study	ISH
FeChPV_probe 4	ACTTTTTCCAGTATTACTACATCCCCAT	−	This study	ISH
FeChPV_probe 5	GCTTTTTCGGCTAATTCAGGGC	−	This study	ISH

### Genome sequencing and phylogenetic analysis

To obtain the genomic sequence of FeChPV in this cat shelter system, primers targeting VP1 and NS1 (Table 1) were designed using SnapGene (version 4.2.4). The amplification procedure was as follows: 35 cycles of 30 s at 95°C, 30 s at 55°C, and 2 min at 72°C. After amplification was completed, the PCR products were purified using an agarose gel DNA purification kit (Magen, Guangzhou, China) and sent to Sangon Biotech for Sanger sequencing. If a double peak was present in the sequence, the PCR product was ligated to the pClone-007 simple vector (Tsingke Biotech, Beijing, China), and several clones were sequenced to identify each sequence. Finally, the sequences of VP1 and NS1 were spliced to obtain a nearly full-length FeChPV genome, and the sequences obtained in this study were deposited in GenBank.

All FeChPV genome sequences from previous reports from Canada and Italy were obtained from the GenBank database (Li et al., 2020; Di Profio et al., 2022). To construct the phylogenetic tree, sequences of chaphamaparvoviruses prevalent in other species were also downloaded from the NCBI website, and protoprovirus was selected as the outgroup to identify the roots. Multiple comparisons of nucleic acid sequences were performed using the ClustalW method, and then the tree was constructed with the neighbor-joining method with 1,000 bootstrap replications in MEGA11 (version 11.0.10; Tamura et al., 2011).

### Hematoxylin–eosin staining and *in situ* hybridization

Necropsy was performed on the cat (#7) that died after treatment failure. Nasal turbinate, cervical lymph gland, brain, and spleen tissues were collected for histopathological examination. The tissue blocks were fixed in 10% phosphate-buffered formalin. Twenty-four hours later, the tissue was dehydrated and embedded in paraffin, sectioned, and then stained with Hematoxylin–eosin (HE) (Servicebio, China).

For the *in situ* hybridization (ISH) assay, SweAMI probes targeting the FeChPV NS1 gene were designed (Table 1) and incubated with sections of nasal turbinate, lymph node, brain, and spleen tissues. The specific methodological steps were carried out with reference to a previous study (Li et al., 2013). After HE staining and ISH assays, the sections were observed under a light microscope (Leica, Germany).

## Results

### Clinical sample detection

**Figure 1:**
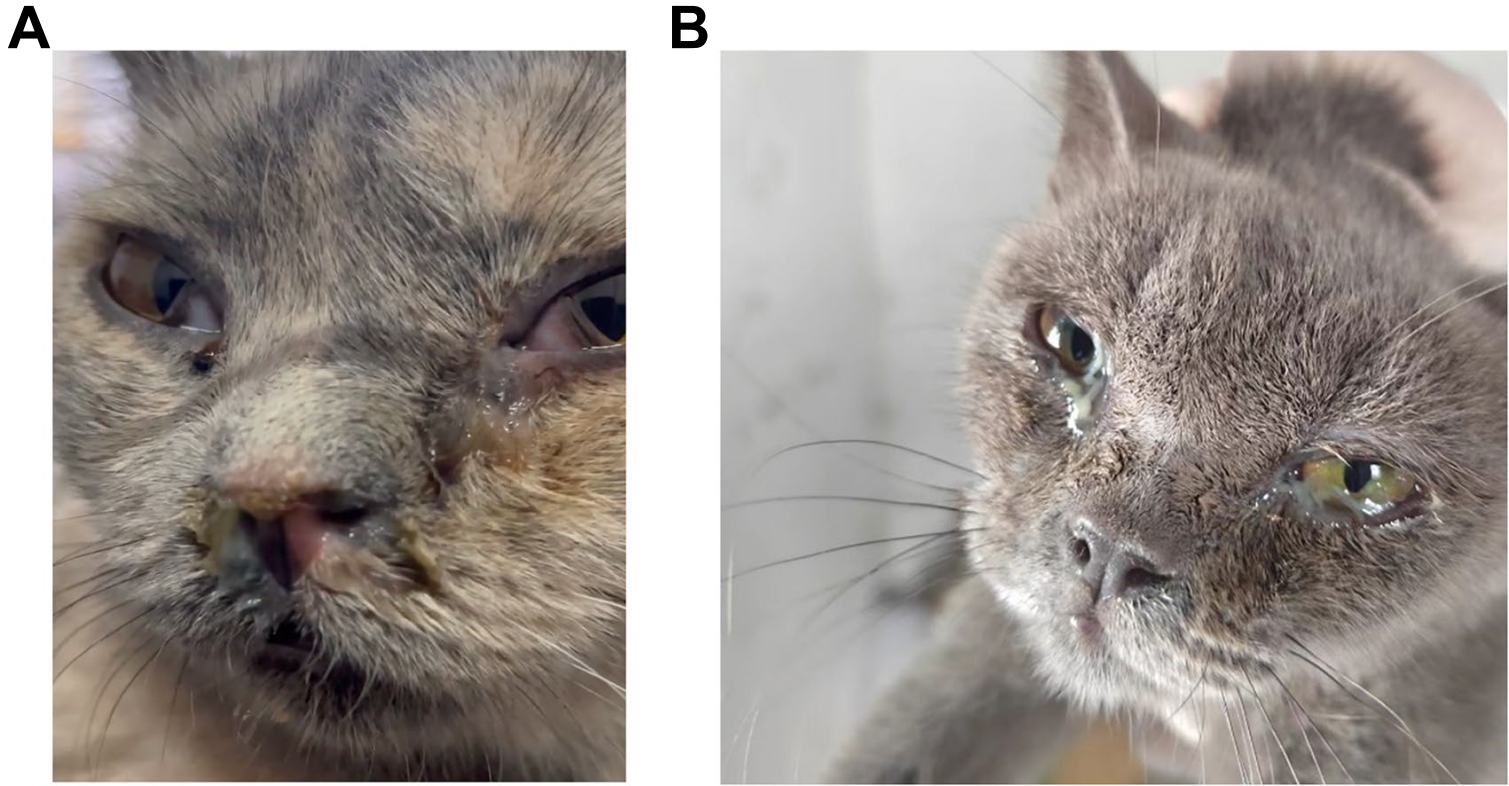
Clinical signs of the cats in the shelter system. **(A,B)** FeChPV-positive cat #9 had respiratory distress, exposed membrana nictitans, and purulent secretions from the eyes and nose. **(B)** FeChPV-positive cat #11 had severe conjunctivitis and increased ocular discharge.

The cats in shelters showed obvious clinical signs, commonly including purulent conjunctivitis, purulent eyelid secretions, exposed membrana nictitans and a sickly appearance. Additionally, associated with rhinitis, the cats exhibited purulent discharge from the nose and labored breathing (Figure 1). Among the cats, cat #7 developed a head tilt, suggesting neurological involvement, before death. Finally, eye and nose swabs were collected from all 37 cats in the cat shelter for virus detection. FeChPV was positive by general PCR in 30/37 (81.08%) samples from cats; 24/30 (80%) FeChPV-positive cats were coinfected with more than one other pathogen, including FCV, FPV, *C. felis*, *M. felis* or FHV-1 (detected by PCR or RT–PCR). However, *M. felis* and IAV were not detected in this investigation (Supplementary Table S1). Unfortunately, although cat #7 was infected with only FeChPV and was treated quickly, its health condition gradually deteriorated until it died. In summary, in this cat shelter outbreak, FeChPV has been spreading in cats and leading to serious symptoms.

### Pathological necropsy and distribution of viral DNA in tissues

**Figure 2:**
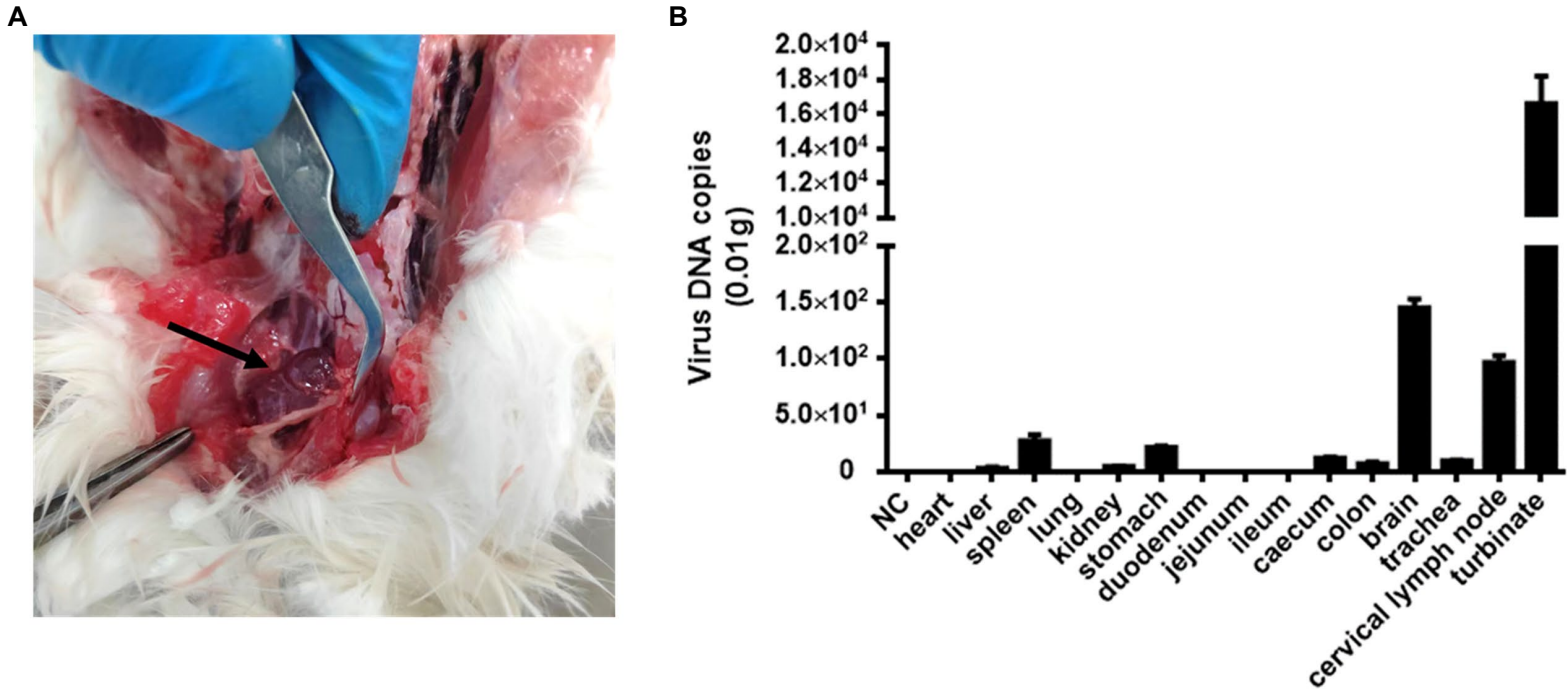
Pathological autopsy and tissue distribution of viral DNA. **(A)** Cats #7 with enlarged and bleeding lymph nodes in the neck. **(B)** The virus DNA loads of FeChPV in each tissue *in vivo*.

Cat #7 died and subsequently underwent general necropsy. The pathological examination showed meningeal hemorrhage with edema and enlarged cervical lymph glands with hemorrhage (Figure 2A). Increased nasal secretions, nonsignificant lung changes, and inflammation and necrosis of the spleen were observed. Interestingly, there were no significant lesions in the kidneys, liver, or digestive organs (data not shown). Subsequently, DNA copies of FeChPV within the tissue samples were detected. The results showed that the nasal turbinate contained the most significant amount of FeChPV DNA, followed by the brain, cervical lymph node, spleen tissues, stomach, cecum, tracheal tissues, colon, kidney, and liver (Figure 2B). It is therefore hypothesized that the virus replicates mainly in the nasal turbinate, brain cells and lymphoid tissue, and the virus replicated in brain tissue may be related to neurological symptoms in cats.

### HE and ISH analysis

**Figure 3:**
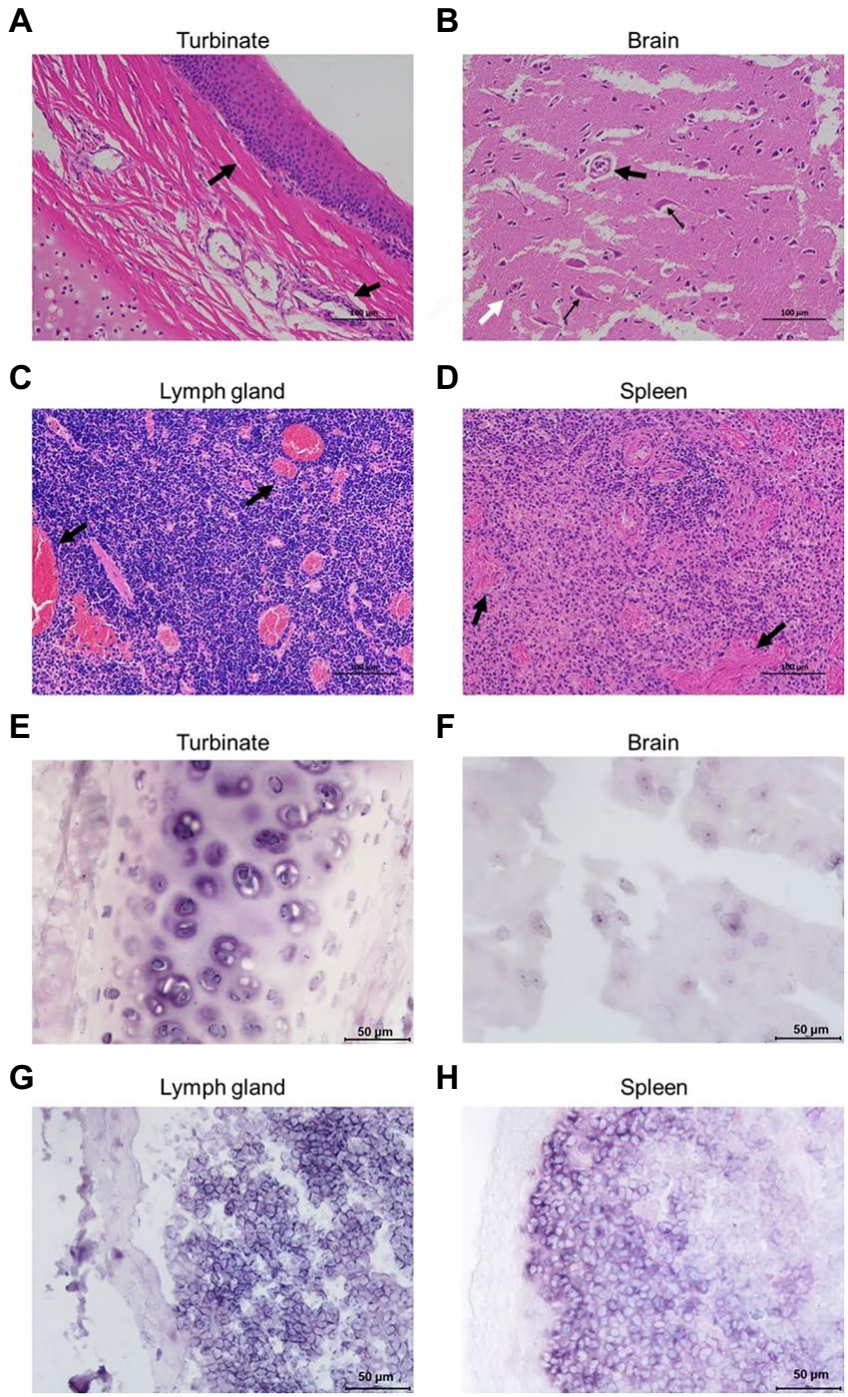
Pathological analyses of the FeChPV-infected cats. **(A)** HE staining of nasal turbinate tissue from cat #7, with infiltration of inflammatory cells in the mucosal layer cells. **(B)** Generalized edema and necrosis of the brain tissue. Neuronal vacuole degeneration and atrophy (small black arrows) with satellitosis (large black arrows) and neuronophagia (white arrows). **(C)** Diffuse proliferation of lymphocytes in the lymph nodes, with severe vascular congestion, manifesting as lymphadenitis. **(D)** Spleen tissue with congestion and lymphocytic infiltration. **(E–H)** Each tissue sample was positive for FeChPV by ISH analysis, and the viral nucleic acid target is shown in blue–purple.

Formalin-fixed nasal turbinate, brain, lymph node, and spleen tissues from Cat #7 were stained for HE analysis. The results showed infiltration of inflammatory cells in the mucosal layer of the nasal turbinate tissue (Figure 3A). Notably, the brain tissue was generally edematous and necrotic, with cavities appearing around the cells due to the collection of edematous fluid. Neuronal vacuolation degeneration, atrophy, satellitosis and neuronophagia were observed (Figure 3B). Diffuse proliferation of lymphocytes and swelling and congestion of lymph node vessels manifested as congestive proliferative lymphadenitis (Figure 3C). The spleen tissue was congested and hemorrhagic, with lymphocyte infiltration (Figure 3D).

Abundant viral nucleic acids were detected in the nasal turbinate and brain tissues of cat #7 by ISH analysis (Figures 3E,F). Nucleic acid positivity was also demonstrated in the lymph node and spleen tissues (Figures 3G,H), suggesting that nasal turbinate and brain tissue cells and even immune cells may be the target cells of FeChPV.

### Genome amplification and phylogenetic analysis

Eye and nose swab samples from cat #7 (dead), cat #8 (dead, coinfected with FCV), and cat #36 (severely ill but survived) were collected for genome amplification. Interestingly, we observed two double peaks in the sequencing of the PCR product of cat #8-derived FeChPV. To determine the correct assembly, additional a pair of primers were designed on either side of the gene region containing the mutation. Subsequently, the PCR product of this sample was ligated to the pClone007 vector, and several clones were selected and sequenced. Eventually, two different mutations of the viral genome, named FeChPV C8-1/22/CHN and C8-2/22/CHN, were identified in the sample from cat #8. After the sequences were spliced, we obtained nearly full-length genome sequences (3,442 bp) of four isolates from three cat samples; the genomes were uploaded to the GenBank database, and accession numbers were obtained (OP499830-OP499833).

**Figure 4:**
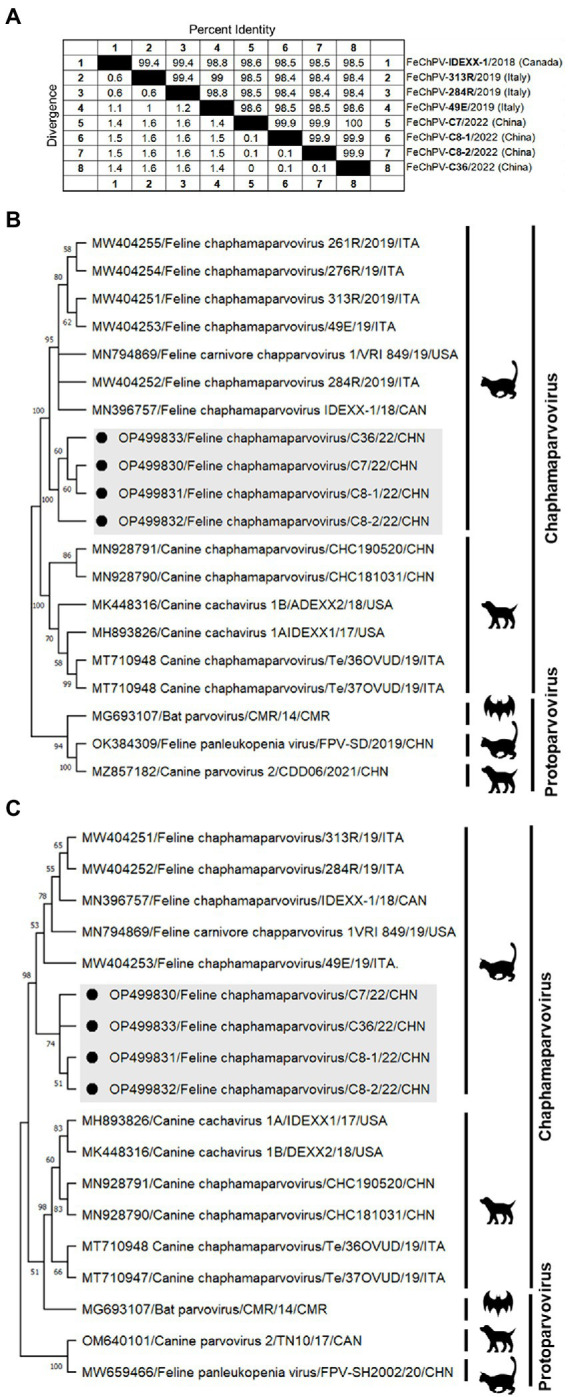
Genome amplification and phylogenetic analysis. **(A)** Genomic homology analysis of the four FeChPV isolates in this study and other isolates. **(B,C)** Neighbor-joining trees based on the nucleotide sequences of the VP1 and NS1 genes. The two trees were constructed using the neighbor-joining method performed in MEGA 11 (version 11.0.10) with 1,000 replicate bootstrap replicates (p-distance model). The black dots indicate the isolates identified in this study.

**Figure 5:**
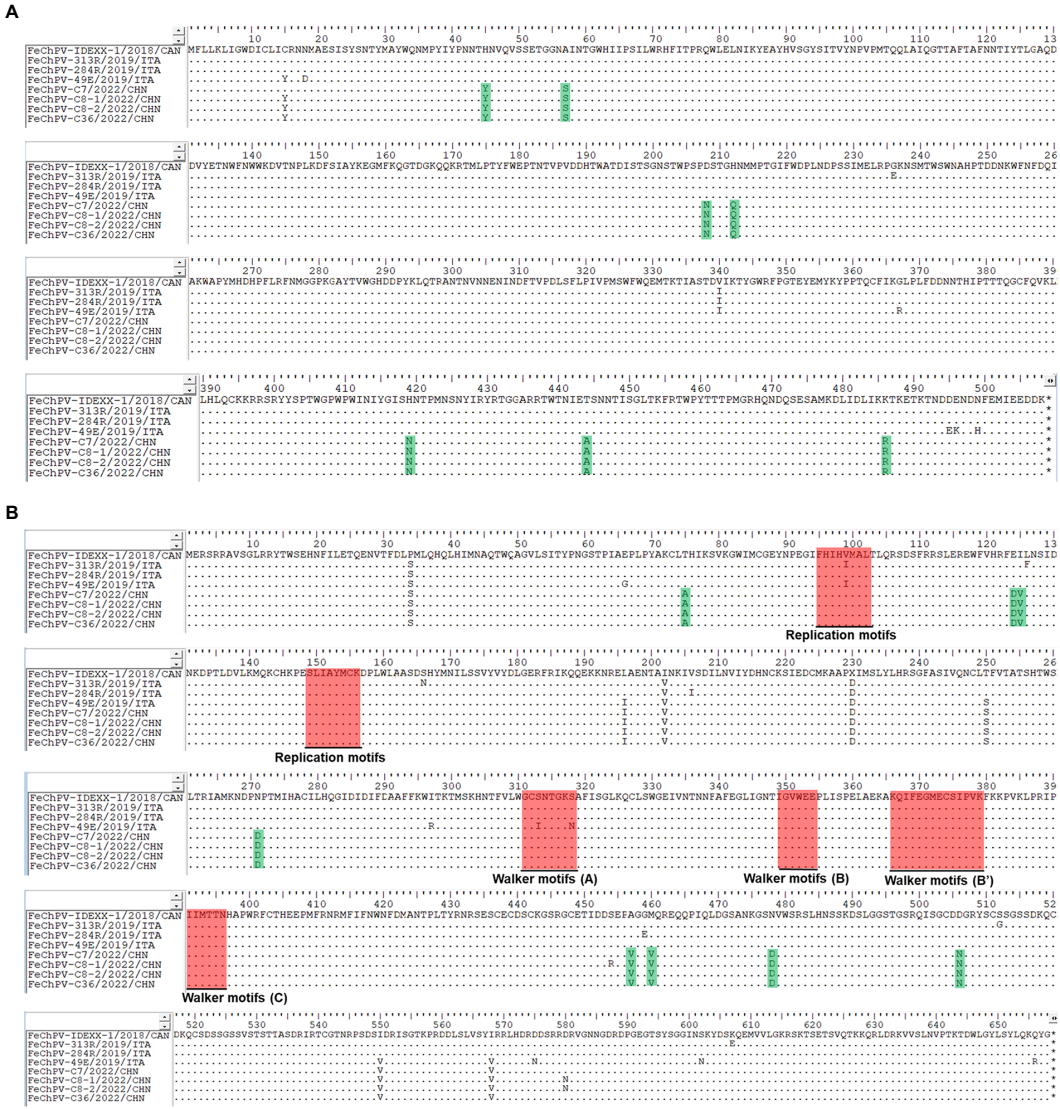
Amino acid mutations in VP1 and NS1 proteins. Amino acid analysis of the VP1 protein **(A)** and NS1 protein **(B)** of the four Chinese FeChPV strains compared with the representative Canadian and Italian strains. The unique amino acid mutation sites of the viral proteins of the four Chinese FeChPV strains were highlighted in green. The conserved motifs of the NS1 protein were labeled using a red background.

Homology analysis showed that the FeChPV strain in this study had 98.4–98.6% genomic homology with the Canadian and Italian strains (Figure 4A), indicating a very close relationship. To further understand the phylogeny of FeChPV in China, we constructed neighbor-joining trees (p-distance model, bootstrap value = 1,000) based on the reference strain and the nucleotide sequences of the VP1 gene and NS1 gene of FeChPV. The results showed that in the VP1-based tree (Figure 4B), the prevalent ChPVs in cats and dogs formed distinct clusters. The four FeChPVs in this study formed a small evolutionary branch of their own, while the strains found in Canada and Italy were clustered in another small branch, although all FeChPVs were in a large evolutionary branch. Interestingly, the evolutionary tree based on the NS1 gene also showed similar clustering features (Figure 4C). The four FeChPV strains in this study clustered into an independent branch, suggesting that they may represent a novel FeChPV genotype. In addition, the amino acid mutations in VP1 and NS1 between the different strains were analyzed. VP1 encodes 508 amino acids, and NS1 encodes 658 amino acids. The results showed that seven unique amino acid mutation sites were present in VP1 (Figure 5), while eight unique amino acid mutation sites were present in NS1 (Figure 5). These unique mutations may be associated with URTD, as the capsid proteins of parvovirus tend to affect viral and host receptor binding, while NS1 is associated with viral replication. Mutations did not appear in the replication initiator (endonuclease) motifs (^95^FHIHV/IMAL^102^ and ^159^SLIAYMCK^156^) of the NS1 protein (Ilyina and Koonin, 1992), and no change was seen in the conserved Walker motifs (Walker A-^311^GCSNTGKS^318^, Walker B-^349^IGVWEE^354^, Walker B′-^366^KQIFEGMECSIPVK^379^, and Walker C-^391^IIMTTN^396^) of the of the helicase domain (Figure 5; Walker et al., 1982; Di Profio et al., 2022).

## Discussion

In this study, we analyzed the first detection of FeChPV in a cat shelter in Guangzhou, China. Cats in the shelter exhibited URTD, with an infection rate of 81.08% (30/37), indicating widespread transmission of the virus in the cat shelter. In addition, coinfection with FCV, FPV, *C. felis*, *M. felis*, or FHV-1 was observed in this feline population. To date, reports on the pathogenicity of ChPVs in vertebrates remain very limited. Mouse kidney parvovirus has been shown to cause inclusion body nephropathy (Roediger et al., 2018). ChPV in the paradise tanager is associated with the development of nonsuppurative encephalitis and neuritis (Roediger et al., 2018). ChPV in black bears has been reported to be associated with nonsuppurative encephalitis (Alex et al., 2020). Although FeChPV was previously shown to be present in healthy cats (2/70) in a study in Turkey (Abayli and Can-Sahna, 2022), its pathogenic potential cannot be ruled out. In this study, FeChPV without FCV or FHV-1 coinfection was detected in one of the dead cats (cat #7). FCV and FHV-1 are the main viral pathogens of URTD in cats, with FHV-1 causing rhinotracheitis and FCV often causing stomatitis and gingivitis (Liu C. et al., 2020). Larger-scale investigations and animal regression experiments are needed to determine the pathogenic capacity of FeChPV.

The cats in this shelter exhibited severe clinical signs, such as coughing, runny nose, and even neurological symptoms. Notably, we did not observe any cats with severe diarrhea symptoms in this outbreak, although previous studies have suggested that FeChPV is associated with diarrhea (Di Profio et al., 2022). Interestingly, our study was consistent with previous reports that viral sequences were obtained from upper respiratory tract samples (Di Profio et al., 2022). FeChPV mainly causes respiratory disease symptoms and has a higher viral load in the respiratory organs than in the digestive organs. Therefore, we speculate that this FeChPV currently found in Guangdong Province relies mainly on respiratory transmission. In addition, necropsy of the cat showed congested and enlarged lymph nodes, meningeal hemorrhage, and marginal infarction of the spleen. Moreover, viral DNA was detected in most tissues, such as the nasal turbinate, brain, lymph node, and spleen tissues. HE staining and ISH assays suggested that FeChPV infection triggered severe pathological changes, e.g., lymphocyte proliferation, vascular congestion, and cellular degeneration and necrosis. The presence of FeChPV in brain tissue is likely to be the cause of encephalitis and neurological symptoms. Notably, viral infection of cerebellar tissue may also be a common cause of ataxia. Therefore, we suggest that cerebellar tissue also needs to be focused on in future studies on FeChPV. We also speculate that FeChPV likely replicates in the immune organs, causing URTD, encephalitis, and lymphadenitis in cats; this warrants further investigation.

The FeChPV strains in this study ranged from 98.4% to 98.6% homologous to the Canadian and Italian strains, respectively, indicating a very close relationship (Figure 4A). In terms of the phylogenetic tree based on VP1 and NS1, Chinese-origin FeChPV is isolated in a small evolutionary branch (Figures 4B,C), suggesting that these FeChPVs may have a common origin but have evolved relatively independently of one another. In addition, the analysis of amino acid mutations between different strains of VP1 and NS1 revealed seven unique amino acid mutation sites in VP1 and eight unique amino acid mutation sites in NS1. These unique mutations may be associated with URTD. We have tried to predict the three-dimensional structure of VP1 and NS1 using SWISS-MODEL and PyMOL software to show the location of the mutated amino acids. However, conclusive data on structure prediction were not available because they do not have a high structural homology with the currently known proteins, and the coverage was insufficient.

Although the mutation did not appear in the conserved motif of NS1, it is worthwhile to further investigate whether this virus containing multiple mutations has a unique tissue tropism.

Previous studies have shown that the NS1 gene of FeChPV is most closely related to that of CaChPV, with 76.0%–77.0% homology (Penzes et al., 2020). A survey from China showed that CaChPV can be transmitted to cats with low prevalence (1.17%, 2/171; Ji et al., 2020). It is a wary of cross-species transmission of viruses between dogs and cats. We highlight the need to further investigate whether FeChPV has the ability to infect dogs, as FeChPV, once cross-species transmission occurs, might potentially recombine with CaChPV to produce new strains.

Many new ChPVs have been detected in recent years, and these viruses have been proposed as new members of the family *Parvoviridae* (Penzes et al., 2020; Yamkasem et al., 2021; Di Profio et al., 2022; Matos et al., 2022). However, these novel ChPVs and their ability to cause disease in their natural hosts have been poorly studied. Since FeChPV may be highly infectious, further studies are necessary to evaluate the pathogenicity and epidemiological characteristics of this novel feline pathogen. The present study may make a useful contribution to the elucidation of the potential pathogenicity of FeChPV.

## Conclusion

For the first time, we recorded and detected FeChPV in a cat shelter outbreak of URTD in China. The strains identified in this study clustered in a large evolutionary branch with FeChPV isolates previously reported in Canada and Italy. FeChPV was considered to be associated with URTD, lymphadenitis, and viral encephalitis. In conclusion, this study has contributed to elucidating the prevalence, genetic diversity, and potential pathogenicity of FeChPV.

## Data availability statement

The datasets presented in this study can be found in online repositories. The names of the repository/repositories and accession number(s) can be found at: https://www.ncbi.nlm.nih.gov/genbank/, OP499830; https://www.ncbi.nlm.nih.gov/genbank/, OP499831; https://www.ncbi.nlm.nih.gov/genbank/, OP499832; and https://www.ncbi.nlm.nih.gov/genbank/, OP499833.

## Ethics statement

The animal study did not require ethical approval because tissue samples were collected only from dead animals, avoiding unnecessary pain and suffering of the animals.

## Author contributions

XH: conceptualization, methodology, and writing. XH, YL, BC, HW, XW, and XX: investigation. SL and PZ: review and editing, funding acquisition, project administration, and supervision. All authors contributed to the article and approved the submitted version.

## Funding

This project was supported in part by the National Natural Science Foundation of China (31872454) and the Natural Science Foundation of Guangdong Province (2022A1515010733).

## Conflict of interest

The authors declare that the research was conducted in the absence of any commercial or financial relationships that could be construed as a potential conflict of interest.

## Publisher’s note

All claims expressed in this article are solely those of the authors and do not necessarily represent those of their affiliated organizations, or those of the publisher, the editors and the reviewers. Any product that may be evaluated in this article, or claim that may be made by its manufacturer, is not guaranteed or endorsed by the publisher.

## Supplementary material

The Supplementary material for this article can be found online at: https://www.frontiersin.org/articles/10.3389/fmicb.2022.1064747/full#supplementary-material
