# Supplementary material for: Detection of FeChPV in a cat shelter outbreak of upper respiratory tract disease in China
Source: Front Microbiol. 2022 Dec 8;13:1064747. doi: 10.3389/fmicb.2022.1064747 (PMC9773189; doi:10.3389/fmicb.2022.1064747)
Supplement: Supplementary file 1 [file Table_1.docx]

**Table S1.** Background information about the cats and results of multiple pathogens detection.

| No. | Gender | Breed | FeChPV | M.felis | IAV | FCV | C.felis | FHV-1 | FPV |
| --- | --- | --- | --- | --- | --- | --- | --- | --- | --- |
| C1 | Female | British shorthair | + |  |  | + |  |  |  |
| C2 | Female | British shorthair | + |  |  | + |  |  |  |
| C3 | Male | British shorthair | + |  |  | + | + |  |  |
| C4 | Female | Chinese Li Hua | + |  |  |  |  |  | + |
| C5 | Female | Chinese Li Hua | + |  |  | + |  |  |  |
| C6 | Female | Chinese Li Hua |  |  |  | + |  |  |  |
| C7 | Male | British shorthair | + |  |  |  |  |  |  |
| C8 | Male | British shorthair | + |  |  | + |  |  |  |
| C9 | Male | Ragdoll | + |  |  | + |  |  |  |
| C10 | Female | British shorthair | + |  |  |  |  |  |  |
| C11 | Female | British shorthair | + |  |  |  |  |  |  |
| C12 | Female | American shorthair | + |  |  |  |  |  |  |
| C13 | Male | American shorthair | + |  |  |  | + |  |  |
| C14 | Male | American shorthair | + |  |  |  |  |  |  |
| C15 | Male | Chinese Li Hua | + |  |  | + |  |  |  |
| C16 | Male | Chinese Li Hua | + |  |  |  | + |  |  |
| C17 | Male | Chinese Li Hua | + |  |  |  |  |  |  |
| C18 | Female | American shorthair | + |  |  | + |  |  |  |
| C19 | Male | Ragdoll | + |  |  | + |  |  |  |
| C20 | Female | British shorthair | + |  |  |  | + |  |  |
| C21 | Female | scottish fold | + |  |  |  |  |  |  |
| C22 | Female | British shorthair | + |  |  |  |  |  |  |
| C23 | Female | Chinese Li Hua | + |  |  |  |  |  |  |
| C24 | Male | British shorthair |  |  |  | + |  |  |  |
| C25 | Female | British shorthair | + |  |  | + | + |  |  |
| C26 | Male | British shorthair | + |  |  |  |  |  |  |
| C27 | Female | British shorthair | + |  |  |  |  |  |  |
| C28 | Male | Chinese Li Hua | + |  |  |  | + |  |  |
| C29 | Female | Chinese Li Hua | + |  |  |  |  |  |  |
| C30 | Female | British shorthair |  |  |  |  |  |  |  |
| C31 | Male | British shorthair | + |  |  | + |  |  |  |
| C32 | Male | Chinese Li Hua |  |  |  |  |  |  |  |
| C33 | Male | British shorthair |  |  |  |  | + |  |  |
| C34 | Female | British shorthair |  |  |  |  | + |  |  |
| C35 | Male | British shorthair |  |  |  |  |  |  |  |
| C36 | Male | Chinese Li Hua | + |  |  |  | + |  |  |
| C37 | Male | Chinese Li Hua | + |  |  |  |  | + |  |

“+” means the result was positive for target pathogen.
