# Supplementary material for: Detection of FeChPV in a cat shelter outbreak of upper respiratory tract disease in China
Source: Front Microbiol. 2022 Dec 8;13:1064747. doi: 10.3389/fmicb.2022.1064747 (PMC9773189; doi:10.3389/fmicb.2022.1064747)
Supplement: Supplementary file 2 [file Table_2.docx]

**Table S2**. List of primers for detection other pathogenes.

| Oligonucleotide | Sequence (5′–3′) | Sense | Reference | Use |
| --- | --- | --- | --- | --- |
| FCV_F | AACCTGCGCTAACGTGCT | + | Zhou et al. (2020) | Diagnostic PCR |
| FCV_R | TGWATTCCCATGTAGGAGGC | - | Zhou et al. (2020) | Diagnostic PCR |
| FPV_F | AAGACGTGCAAGCGAGTCC | + | Zhou et al. (2020) | Diagnostic PCR |
| FPV_R | GAGCGAAGATAAGCAGCGTAA | - | Zhou et al. (2020) | Diagnostic PCR |
| M.felis_F | GGAATAACGGTGAGAAAT | + | This study | Diagnostic PCR |
| M.felis_R | AGTTTACAACCCGAAGG | - | This study | Diagnostic PCR |
| C.felis_F | TTGTCGGATTGATTGGTCTT | + | Zhou et al. (2020) | Diagnostic PCR |
| C.felis_R | AGTTGGGTTCCAGGTTGTTA | - | Zhou et al. (2020) | Diagnostic PCR |
| FHV-1_F | TACTTCAAGCCTTACGACCACG | - | Zhou et al. (2020) | Diagnostic PCR |
| FHV-1_R | GATCGAGACCTCTTTACCCTCA | - | Zhou et al. (2020) | Diagnostic PCR |
| IAV_F | CAGAGACTKGAARRTGTCTTTGC | - | Zhou et al. (2020) | Diagnostic PCR |
| IAV_R | CTACGCTGCAGTCCTCGCTC | - | Zhou et al. (2020) | Diagnostic PCR |
